# Skeletal muscle memory: implications for sports, aging and nutrition

**DOI:** 10.3389/fnut.2025.1701520

**Published:** 2025-11-19

**Authors:** Íñigo M. Pérez-Castillo, Sergio R. Ruiz-Caride, Ricardo Rueda, José López-Chicharro, Felipe Segura-Ortiz, Hakim Bouzamondo

**Affiliations:** 1 Abbott Nutrition, R&D, Granada, Spain; 2 Real Madrid, Medical Services, Madrid, Spain; 3 Abbott Nutrition, R&D, Chicago, IL, United States

**Keywords:** myonuclei, satellite cells, detraining, retraining, athlete, hypertrophy, aging, nutrition

## Abstract

Observations of an enhanced hypertrophic response in previously trained muscles following periods of detraining have led researchers to propose that muscle tissue retains a form of “cellular memory”, even after returning to baseline muscle mass. Recent advances in research methodologies have enabled deeper investigation into the underlying mechanisms, with myonuclear permanence emerging as a potential candidate. While intracellular signaling pathways that mediate anabolic stimuli and promote translational efficiency have been extensively studied, the role of transcriptional output, governed by the number of myonuclei, remains comparatively underexplored. A solid body of evidence in humans supports the need for satellite cell-mediated myonuclear accretion to achieve muscle growth that exceeds the transcriptional limits of existing myonuclei. However, it remains unclear whether accrued myonuclei persist indefinitely or are eventually removed, and the mechanisms governing their potential removal remain speculative. Notably, aging populations may not only exhibit diminished capacity to recruit satellite cells but also potentially reduced ability to retain myonuclei, which may be linked to age-related muscle wasting. Training exercise combined with nutritional strategies leveraged by athletes, including protein/amino acid intake, polyphenol-rich ingredients, and different ergogenic compounds such as creatine, might be beneficial to promote satellite cell responses and myonuclear accretion in situations where muscle mass regain and maintenance are purposed, such as injury recovery and aging.

## 1 Introduction

Skeletal muscle hypertrophy refers to the increase in the size of muscle tissue largely due to an increment in myofiber size rather than number. It occurs in situations when muscle protein synthesis (MPS) exceeds muscle protein breakdown (MPB), resulting in positive net protein balance throughout cumulative periods (1). Major factors contributing to muscle hypertrophy include protein intake and mechanical overload (2). The increase in cell size rather than cellular count stems from two key physiological factors. First, skeletal muscle cells are, almost exclusively, postmitotic, so that their capacity for replicating their DNA and dividing is blunted (3). The other factor relates to their unique morphology, as they are long, tubular multinucleated cells (3). The larger size of these cells comes with increased cytoplasm and organelle content, therefore requiring higher transcriptional output to satisfy their structural demands, which is met by the numerous nuclei present in each myofiber. Early studies from the 1970s described the differential arrangement of myonuclei within skeletal muscle fibers (4). Later experiments led to propose the concept of myonuclear territory or domain—a finite volume of cytoplasm that a single myonuclei transcriptionally supports (5). Accordingly, new myonuclei would be needed for sustaining hypertrophy upon exceeding the “ceiling” of existing myonuclear domains. It is only recently that advancements in experimental methodologies, such as cell cultures, cellular reporters, RNA sequencing platforms, and genetic labeling and tracking techniques (6), have revisited such hypotheses and deepened our understanding of the mechanisms involved in hypertrophy, atrophy, and the role of myonuclei and their precursor cells, satellite cells.

To elucidate whether satellite cells contribute to sustained hypertrophy by supplying new myonuclei once a myonuclear domain “ceiling” is reached, versus their role solely in muscle regeneration in response to hypertrophic stimuli, preclinical models have offered valuable mechanistic insights. In rodent models, anabolic paradigms typically include synergistic ablation surgery, electrical stimulation, and, more recently, weight-loaded ladder climbing, and progressive weighted-wheel running (PoWeR). While the former involves surgical removal of the *gastrocnemius* and *soleus* to induce functional overload of the *plantaris* muscle (7), weight-loaded ladder climbing and PoWeR are considered to better approximate the effects of muscle loading/hypertrophic stimuli in humans (8, 9). Hypertrophy may also be achieved through endocrine mechanisms involving supraphysiological levels of anabolic androgenic steroids (AAS) and growth factors [e.g., testosterone (10), insulin-like growth factor-1 (IGF-1) (11)], or depletion of well-described negative regulators of muscle mass, such as myostatin (12). Preclinical studies leveraging these methods in adult rodent models with conditional satellite cell depletion have documented mixed findings (detailed later on). Some studies report that muscle hypertrophy can occur without significant myonuclear accretion (13), whereas others have indicated that satellite cell depletion impairs fiber hypertrophy (14). Differences in methodological aspects, including variations in the age of the animal assessed, may contribute to these discrepancies. Nonetheless, emerging preclinical evidence suggests that satellite cells may also contribute to muscle adaptation in adult stages of life through additional mechanisms beyond myonuclear addition, such as regulation of the extracellular matrix (ECM) and epigenetic modulation (13, 15).

Although preclinical models are invaluable for uncovering the cellular and molecular mechanisms governing satellite cell dynamics and myonuclear accretion, caution is warranted when extrapolating these findings to humans. Clinical studies commonly utilize resistance exercise but also incorporate aerobic or concurrent exercise protocols together with protein/amino acid intake to promote muscle growth and study myonuclear physiology and satellite cell dynamics. Results from studies leveraging these protocols suggest that myonuclear domains are not “rigid,” but rather “flexible” and fiber type-dependent (16), with oxidative type I fibers typically having smaller and denser myonuclear domains compared to glycolytic type II fibers (17). This flexibility implies that resident myonuclei—those originally present within a myofiber prior to hypertrophic stimulation—possess transcriptional reserve capacity, allowing them to support hypertrophy, at least to some extent, after both short- and long-term training stimuli without the immediate need of accrued myonuclei—new nuclei derived from satellite cells that have been activated and fused with the myofiber in response to hypertrophic stimuli. However, systematic analyses of clinical studies have reported consistent myonuclear addition when hypertrophy exceeds certain expansion upper limits (e.g., ≈22%) independent of fiber type, sex, and age (18), thus supporting a role of satellite cells in sustaining skeletal muscle hypertrophy in humans.

One area of great scientific interest consists of the long-lasting effects of skeletal muscle hypertrophy following subsequent atrophy/detraining and return to baseline muscle mass conditions. Whereas a role of satellite cells in muscle hypertrophy in the absence of damage remains debated, higher baseline myonuclear content has been linked to a more potent hypertrophic response in animal studies after skeletal muscle atrophy, which may be important to support muscle mass preservation and expansion (19, 20). Interestingly, breakthrough preclinical evidence has supported a certain degree of permanence of myonuclei populations gained with hypertrophy following muscle mass loss, which led authors to speculate that muscle hypertrophy imprints “memory” in the muscle cell (19). This may facilitate a further adaptation to later muscle regrowth, being such myonuclear permanence a potential mechanism involved (19). Although most evidence on this phenomenon emerges from animal studies, recent longitudinal trials conducted in young adults have clearly shown increased myonuclear number after a training period, which is almost entirely maintained during 16 weeks of detraining despite a decrease in fiber size and mass (21). These findings should be cautiously interpreted, as evidence on myonuclear permanence following atrophy has been contentious across various research models through the years, and technical limitations (explained later on) hinder drawing definitive conclusions. Nonetheless, myonuclear permanence and a resulting faster “retraining route,” if proven, may have relevant repercussions in sports settings, particularly in situations of detraining due to episodic disuse such as recovery from injury (22). Indeed, this topic has sparked great interest in the sports community due to its potential implications for antidoping regulation purposes, given the potent effects of supraphysiological levels of AAS on myonuclear accretion (23, 24). Not least important, a potential role of resident myonuclei in augmenting the response to anabolic stimuli might prove relevant for strategies aimed to prevent or attenuate muscle wasting associated with aging. Whether populations of myonuclei may endure permanently in aged individuals or whether myonuclei removal is simply delayed compared to different cellular components in response to age-related muscle wasting requires further investigation.

In our view, the biological implications of myonuclear permanence and other mechanisms contributing to long-term muscle adaptation may offer broader insights into hypertrophy than those limited to the context of AAS abuse in athletes. As cited by Traversa, athletes typically consume higher amounts of proteins/amino acids in combination with training exercise, which may imply amplified activation of satellite cells and consistent myonuclear accretion (23). In alignment, combinations of resistance exercise with predominant concentric component and protein intake were reported to increase satellite cell pool in type I and II myofibers, and enhance type II fiber cross-sectional area (CSA) and myonuclei accretion in healthy young men (25). Commonly used ergogenic supplements, such as creatine, have been reported to further enhance the effect of resistance training in satellite cell activation and myonuclear accretion (26). Evidence on myonuclear permanence might further support an enduring effect of exercise and nutritional interventions aimed to promote myonuclear accretion, which in turn might be beneficial for facilitating regains of muscle mass after extended detraining periods as well as preserving muscle mass with aging. To our knowledge, these aspects have not been reviewed to date. The objectives of this work are to re-evaluate the role of myonuclear accretion in muscle hypertrophy and to examine evidence supporting the concept of muscle memory. We also explored implications and research opportunities in the contexts of detraining and aging, and discussed how nutritional strategies commonly employed by athletes may be leveraged by aged individuals to support muscle mass.

## 2 Mechanisms underlying skeletal muscle hypertrophy

**Figure 1:**
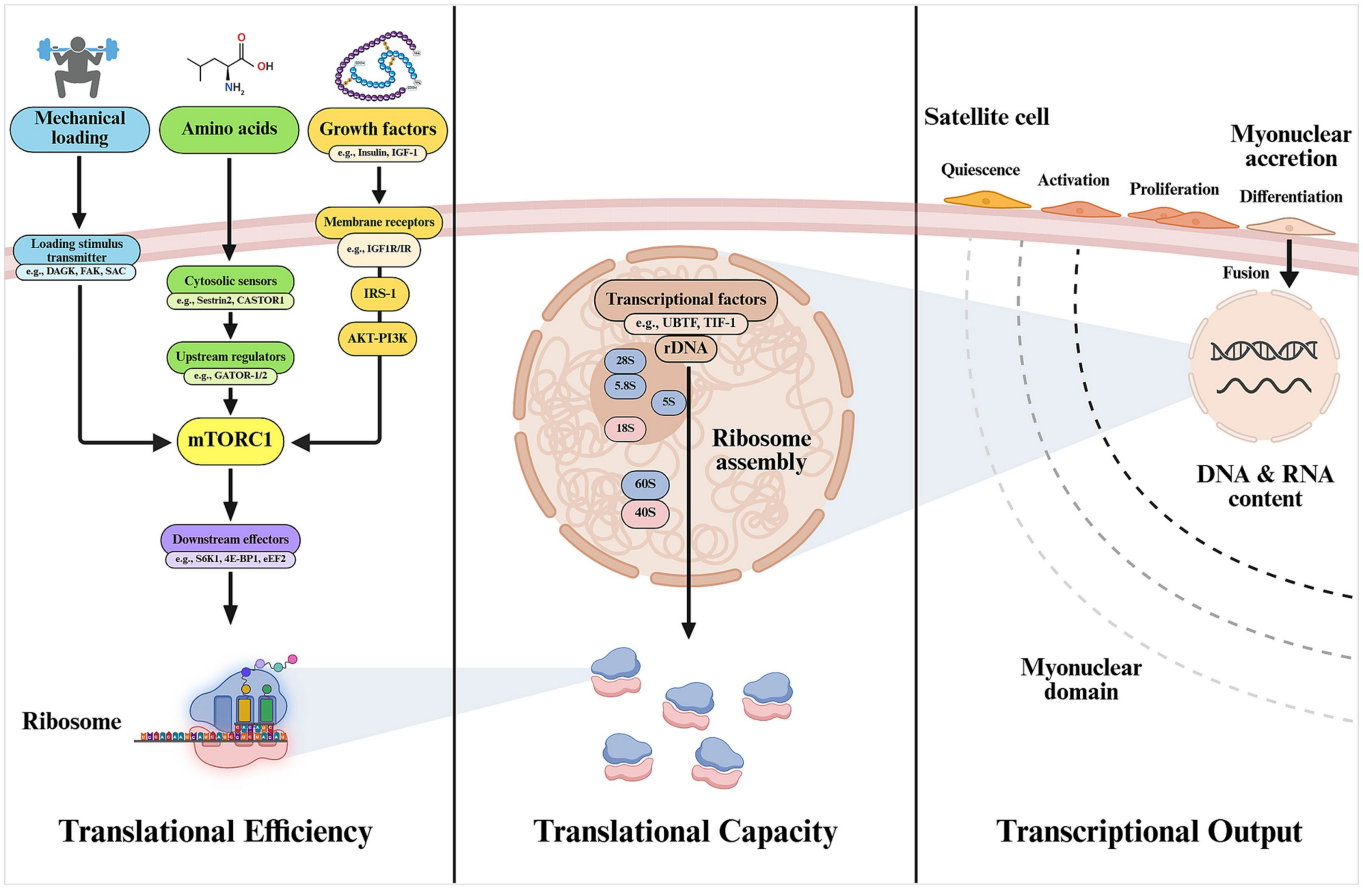
Main cellular mechanisms involved in muscle protein synthesis. Translational efficiency, although not exclusively, is largely determined by the activation of mTORC1 through existing functional ribosomes. The total pool of ribosomes will reasonably determine the maximum synthesis capacity (translational capacity) of the muscle cell. Myonuclear accretion through activation, proliferation, and fusion of satellite cells is proposed to be needed to support the increased transcriptional needs of the growing muscle and maintain a physiologically manageable myonuclear domain, ensuring efficient regulation of gene expression (transcriptional output) for protein synthesis. 4E-BP1, eukaryotic translation initiation factor 4E-binding protein 1; CASTOR1, cytosolic arginine sensor for mTORC1 subunit 1; DAGK, diacylglycerol kinase; FAK, focal adhesion kinase; GATOR1/2, GTPase-activating protein activity toward RAGs 1/2; IGF-1, insulin-like growth factor-1; eEF2, eukaryotic elongation factor 2; IR, insulin receptor; IRS-1, insulin receptor substrate-1; mTORC1, mechanistic target of rapamycin complex 1; SAC, stretch-activated channel; S6K1, ribosomal protein S6 kinase-1; Sestrin2, leucine sensor for mTORC1 pathway; TIF-I, transcription intermediary factor 1; UBTF, upstream binding transcription factor. Created with BioRender.com.

An increase in muscle size results from the activation of the cellular translational machinery through the activity of existing ribosomes, referred to as “translational efficiency,” in response to anabolic stimuli, and may be mirrored or dictated by changes in transcriptional output, which is driven by myonuclear content (27), and by changes in ribosome number, known as “translational capacity” (28). A simplified scheme summarizing major processes involved in MPS is represented in Figure 1.

Changes in muscle protein synthesis can occur rapidly, within minutes to hours, due to regulatory mechanisms that enhance mRNA translation in response to anabolic stimuli (29). Translational machinery is largely governed by the activity of the mechanistic target of rapamycin complex 1 (mTORC1). The molecular basis of mTORC1 and its physiological regulation by nutrients, mechanical loading, and hormones has been comprehensively reviewed elsewhere (30, 31). Briefly, protein amino acids, often considered the “building blocks” of the muscle, provide structural and functional support to the cell. Amino acids are detected by cytosolic sensors such as Sestrin2, in the case of leucine (32), and cytosolic arginine sensor for mTORC1 subunit 1 (CASTOR1) for arginine (33) which, through modulating interactions with different mTOR regulators [e.g., GTPase-activating protein activity toward RAS-related GTPases 1/2 (GATOR-1/2)], facilitate the recruitment of mTORC1 to the lysosome and enable the interaction with its kinase activator RAS homolog enriched in brain (Rheb) (34). This process is key for enabling mTORC1 kinase activity over major downstream substrates such as ribosomal protein S6 kinase-1 (S6K1) and the repressor eukaryotic translation initiation factor 4E-binding protein 1 (4E-BP1) which, in turn, promotes translation initiation and elongation at the ribosomal level (2), thus facilitating the synthesis of new proteins.

Activation of mTORC1 signaling also underlies the activity of certain growth factors on muscle anabolism, being IGF-1 and insulin among the most extensively researched. These bind to the insulin/IGF-1 receptors on the sarcolemma and facilitate the phosphorylation of the insulin receptor substrate 1 (IRS-1), resulting in recruitment and activation of phosphoinositide 3-kinase (PI3K). In turn, PI3K produces phosphatidylinositol-3,4,5-trisphosphate (PIP3), a docking molecule for Akt and phosphoinositide-dependent kinase 1 (PDK1), resulting in Akt phosphorylation by the latter. Akt is a key kinase that modulates the activity of mTORC1 through phosphorylation of the negative regulator tuberous sclerosis complex 2 (TSC2) (2). Importantly, Akt also inhibits forkhead box O (FoxO) transcription factors, especially the muscle-specific forms FoxO1 and FoxO3. This modification causes the FoxO proteins to move from the nucleus to the cytoplasm, thereby suppressing the expression of genes that activate the ubiquitin-proteasome system (UPS), including key E3 ubiquitin ligases like muscle RING finger 1 (MuRF1) and atrogin-1/muscle atrophy F-box gene (MAFbx), as well as genes involved in autophagy and lysosomal degradation pathways, which is particularly relevant for the inhibition of muscle catabolism (35).

It should be noted that PI3K-Akt–mTOR signaling is also suggested to participate in the catabolic effects of endocrine negative regulators of skeletal muscle mass. Members of the transforming growth factor beta (TGF-β) superfamily, such as activin A and myostatin, are well-known inhibitors of MPS. They primarily exert their effects by binding to activin type II receptors (ActRIIA/IIB), which subsequently recruit and activate type I receptors, such as activin receptor-like kinases 4/5 (ALK4/5). This activation leads to the phosphorylation of Smad elements (Smad 2/3), which promotes complex formation with Smad 4 and its translocation to the nucleus to regulate the expression of target genes (36). ActRIIA/IIB–ALK4/5–Smad2/3/4 signaling might also interact with Akt as indicated by promoted skeletal muscle hypertrophy via mTOR signaling following myostatin inhibition (37). In the same vein, several TGF-β proteins are inhibited by follistatin, an endogenous molecule considered a pro-hypertrophic factor (38). Nonetheless, different members of the TGF-β superfamily have been shown to induce muscle hypertrophy in animal models through activation of different Smad elements (Smad1/5/8) and crosstalk with Akt/mTOR, being bone morphogenetic proteins (BMPs) the most notorious examples (39).

Androgens, particularly testosterone and active byproducts such as dihydrotestosterone (DHT), are major players in muscle anabolism through genomic and non-genomic mechanisms. Genomic effects result from binding to the nuclear androgen receptor (AR) in the cytoplasm, resulting in nuclear translocation through dissociation from the AR-heat-shock protein (HSP) complex and binding to androgen response elements (ARE), which, in combination with transcription factors, facilitates the upregulation of genes involved in protein accretion and anabolism (40). Additionally, androgens may also act through a membrane-linked mechanism (41) facilitating the activation of Akt/mTOR signaling, which may involve promoted IGF-1 expression (42). In alignment, androgen removal *in vivo* has been linked to impaired myofibrillar protein synthesis through Akt/mTOR signaling, which was shown to be reversed by the administration of nandrolone (43). Naturally, mTOR-mediated hypertrophy is not only limited to the action of endogenous hormones and nutrition, with different pharmacological agents (e.g., β2-agonists) being capable of stimulating protein synthesis through this pathway, a topic that remains out of the scope of the present review (44).

Mechanotransduction refers to the transmission of the mechanical loading stimulus into intracellular biochemical signals involved in protein balance regulation. To date, the complete signaling pathway, starting from the mechanical sensors and culminating in transcriptional and translational modulation, is yet to be completely elucidated (31). Main candidates of mechanical loading sensors facilitating the activation of mTORC1 upon loading stimuli appear to be Akt-independent, and may include transient myocellular increments in phosphatidic acid (PA) through promoted diacylglycerol kinase (DAGK) activity (45), transmembrane integrins and focal adhesion kinase (FAK) signaling (46), and stretch-activated channels (SAC) linked to calcium and sodium influx (47). Mechanotransduction remains an area of active research and mechanical stimuli might also modulate nutrient-related pathways such as amino acid sensing and transport proteins or facilitate the dissociation of mTORC1 from inhibitory regulators (TSC2) (31). Last, it should be noted that different lines of research have provided evidence mTOR-independent mechanisms of skeletal muscle hypertrophy. A well-supported example of these includes extracellular signal-regulated kinase 1/2 (ERK1/2), which is a mitogen-activated protein kinase (MAPK) with many downstream effects supporting muscle anabolism (2).

As discussed to this point, most research to date has focused on elucidating and expanding the mechanisms of translational efficiency in response to anabolic stimuli, which is demonstrated to facilitate rapid skeletal muscle hypertrophy. However, the role of different mechanisms such as increased translational capacity and transcriptional output—determined by the number of functional ribosomes and myonuclei per myofiber, respectively—in sustaining muscle hypertrophy and adaptation has been comparatively underexplored. In terms of translational capacity, it is reasonable to consider ribosome content as a determinant of the upper limit of protein synthesis in cells (48). Activation of mTOR and skeletal muscle hypertrophy, attained through different loading protocols, associate with increased total ribosomal RNA (rRNA) (measured as a proxy of ribosomal content), specific rRNA transcripts, and/or upregulation of key transcriptional regulators [e.g., upstream binding transcription factor (UBTF) and transcription intermediary factor 1 (TIF-I)] in preclinical and clinical studies (48–51). Indeed, inhibition of rRNA transcription or related transcription factors compromises cell growth (52). Further, authors have suggested that ribosomal biogenesis is needed for robust muscle hypertrophy at muscle growth thresholds where no further increase in translational efficiency (e.g., S6K1 phosphorylation status) is observed (51). The impact of ribosomal biogenesis on muscle hypertrophy may be modulated by nutrition as shown in research reporting impairments in MPS with postnatal undernutrition that were largely explained by decreased translational capacity in mice (53). Substantial ribosome degradation and impaired ribosomal biogenesis are shown with muscle atrophy (knee immobilization), and changes in ribosomal biogenesis markers are reported to occur in the early phase of muscle hypertrophy and to be reversed following detraining in recent clinical studies (54, 55). Altogether, these data support that ribosomal biogenesis may precede skeletal muscle hypertrophy and associate with the magnitude thereof. A key question that remains is whether hypertrophy must be accompanied, or even preceded, by increased transcriptional output supported by newly accrued myonuclei, or whether resident myonuclei are sufficient to meet the transcriptional demands of growing muscle.

### 2.1 The contributory role of myonuclear accretion to skeletal muscle hypertrophy

Early observations in the 1960s described associations between DNA content and post-natal muscle fiber growth in rats (56) and ignited the idea that myonuclei can only control a limited cytoplasmic territory or domain. Muscle mass expansion would consequently need of myonuclear accretion achieved through satellite cell activation, proliferation, and fusion. While this is demonstrated to be the case during developmental periods of life, the obligatory role of satellite cells in muscle hypertrophy during adulthood has been long debated (57). Research in humans has demonstrated that satellite cells merge with myofibers in response to resistance training to source new myonuclei to the growing muscle (16, 58–60). Additionally, the abundance of satellite cells following either a single resistance exercise session or prolonged resistance training is linked to the extent of muscle fiber hypertrophy (58, 61, 62). Previously thought to be limited to muscle regeneration following damage, these clinical studies, and different preclinical models explained in the following lines have examined a potential role of satellite cells in facilitating myonuclei recruitment, promoting *de novo* hypertrophy, and preceding radial growth.

In a pivotal study conducted by Egner et al. (14), Pax7-diphtheria toxin subunit A (DTA) mice displaying conditional satellite cell ablation upon tamoxifen treatment were reported to be incapable of mounting functional sustained hypertrophy. This animal model of selective satellite cell ablation overcome limitations of previous models using X-ray irradiation to induce depletion of satellite cells, a protocol that may have wider deleterious effects on muscle hypertrophy mechanisms (63). However, other authors have argued that several methodological limitations including not only the satellite cell depletion model used but also the muscle type studied among other technical aspects might have compromised the accuracy of these results (64). Later studies supported Gundersen lab’s findings and observed that myomaker deletion—a muscle specific membrane protein required for myoblast fusion—in satellite cells leads to a complete reduction of *de novo* hypertrophy in response to mechanical overload (65). Recent research from Gundersen’s group helped further clarify previous controversies. Briefly, experiments on 3D-reconstructed fibers—including both human and mouse fibers—showed that myofibers are DNA scarce in comparison to different cells, fibers growth in parallel to nuclei content, and a permanent increase in myonuclear domain size limits fiber growth (66). In a complementary paper, three independent Myomaker^loxP/loxP^; Pax7^CreER^ mouse models with different myonuclear content (25, 50, 75%) were engineered and authors’ results suggested that myonuclear number is a determinant of skeletal muscle size, and the relationship is not linear with myonuclei having sufficient transcriptional reserve capacity for initial hypertrophy in a range of myofiber flexibility, but myonuclear accretion is ultimately needed for adaptations in adult muscle (27). Also important, preclinical experiments using high-intensity interval training (HIIT) protocols in mice have shed light on the differential role of satellite cells in regeneration from muscle damage from adaptive hypertrophy. Specifically, progressive increases in treadmill incline or speed over 8 weeks were shown to associate with increased myonuclear number at each stage, which also resulted in increased myofiber CSA in the assessed muscles. Adaptive hypertrophy was blunted in absence of myomaker in these animals (67). Interestingly, a recent study using neuromuscular electrical stimulation in plantar flexor muscles of mice observed satellite cell proliferation and myonuclear accretion in absence of overt damage or muscle regeneration in type IIb fibers (68).

Some data discussed above support that satellite cells might not be needed for initial muscle hypertrophy due to resident myonuclei transcriptional reserve; however, transcriptional output needs to increase to support sustained hypertrophy, which is met by myonuclear accretion from satellite cells. Myonuclear domain plasticity may thus be a key component of muscle hypertrophy, as domain size can change with muscle hypertrophy and atrophy, in different species, sex or fiber types (6). Myofibers would utilize two distinct mechanisms to maintain the transcriptional output in line with the increase in cytoplasmatic volume. On one hand, augmenting the transcriptional output of resident myonuclei, as shown by mature mouse myofibers which are suggested to exhibit a significant transcriptional reserve capacity (69). The other proposed mechanism is myonuclear accretion due to satellite cell activation and fusion to myofibers. The balance between both mechanisms is proposed to correlate with muscle fiber size, with smaller myofibers possessing increased transcriptional reserve capacity, and larger fibers requiring satellite cells to fuse and provide new myonuclei (70). Interestingly, a recent study using lineage tracing of nuclei in myofibers elegantly described the transcriptional states of newly fused nuclei and resident pre-existing nuclei concluding that both populations influence each other to maintain growth during skeletal muscle development and adaptation (71).

It should be noted that some clinical experiments conducted by different groups have observed relatively early (5 weeks) increments in myonuclei number with combinations of aerobic and resistance exercise in type I and II fibers compared with resistance exercise alone in young men (60), which would indicate an early contribution of satellite cells to acute hypertrophy. Despite these findings, a role of satellite cells in sustaining long-term muscle hypertrophy is consistent with results from a meta-analysis of 27 studies including a total of 903 participants and investigating changes in muscle CSA and myonuclear content in response to exercise with or without nutritional supplementation or steroid treatment. Findings revealed that ~10% increase in myofiber size leads to a modest rise in myonuclear content, while significantly higher myonuclear number is observed when hypertrophy reaches ~22% increments in fiber size (18). While methodological differences in pooled studies might compromise drawing solid conclusions on specific hypertrophy thresholds, this analysis made evident that myonuclear content can increase with resistance training, supraphysiological levels of AAS, and, potentially, nutritional supplementation.

## 3 Myonuclear permanence and muscle memory

Cessation of anabolic stimuli in situations such as disuse leads to muscle atrophy, which has been classically considered to associate with a loss of different cellular structures including myonuclei. However, the notion that newly accrued or resident myonuclei are removed after the cessation of anabolic stimuli was challenged in a study conducted in 2008 by Bruusgaard and Gundersen (72). Authors explored three different preclinical models of atrophy (denervation, nerve activity blockage, and tenotomy of antagonist muscles) and observed no myonuclear content loss despite clear atrophy and signs of apoptosis, which was linked to removal of nuclei corresponding to satellite cells and stromal cells outside the dystrophin ring (72). In another set of experiments from the same laboratory, authors reported that myonuclear accretion (assessed through labeled nucleotides) preceded CSA increments (6 versus 9 days) in mice subjected to synergistic ablation (73). In alignment with previous findings, myonuclear number was not significantly reduced following 14 days denervation and after 35 days of ablation in spite of decrements in CSA (73). Again, authors observed increased apoptosis during atrophy (terminal deoxynucleotidyl transferase dUTP nick-end labeling (TUNEL) staining), yet only 1 of ≈15,000 screened myonuclei was considered apoptotic (73). In an ensuing study, authors provided further evidence of myonuclear permanence inside dystrophin rings in rats undergoing hindlimb suspension for 14 days (74). In the same fashion, apoptosis was proposed to be confined to nuclei located outside the dystrophin ring (74). Authors postulated that myonuclear number reflects the largest size that the myofiber has ever achieved and that increased myonuclear number is only needed when a fiber grows beyond this maximum size (74). Accordingly, it may be reasoned that further hypertrophy would therefore lead to unlimited increases in myonuclear accretion; however, a ceiling of myonuclear content (plateau) may be reached, which might be speculated to be associated with the ceiling effect observed in hypertrophy protocols. Findings of maintained myonuclei populations were also documented by different research groups (75). Last, Bruusgaard and Gundersen’s laboratory reported findings from a set of experiments conducted in female mice subjected to testosterone administration through pellet implants alone or coupled with muscle overload (19). Steroid treatment alone was sufficient to potentially increase myonuclear number, which was further enhanced when synergistic ablation was applied. Pellet removal (either 3 weeks or 3 months) was not associated with a significant decrease in myonuclei number (19). Probably, the most interesting finding consisted of a higher increment in CSA when introducing overload (14 days) in rats previously treated with steroids compared to the sham group (42% versus 21%) (19). Extending the overloading protocol to 3 months led to similar findings, increasing CSA by 31% in the treated group compared to only 6% in the sham group (19). These findings support the existence of a “retraining route” that might facilitate gains in muscle mass after atrophy.

**Figure 2:**
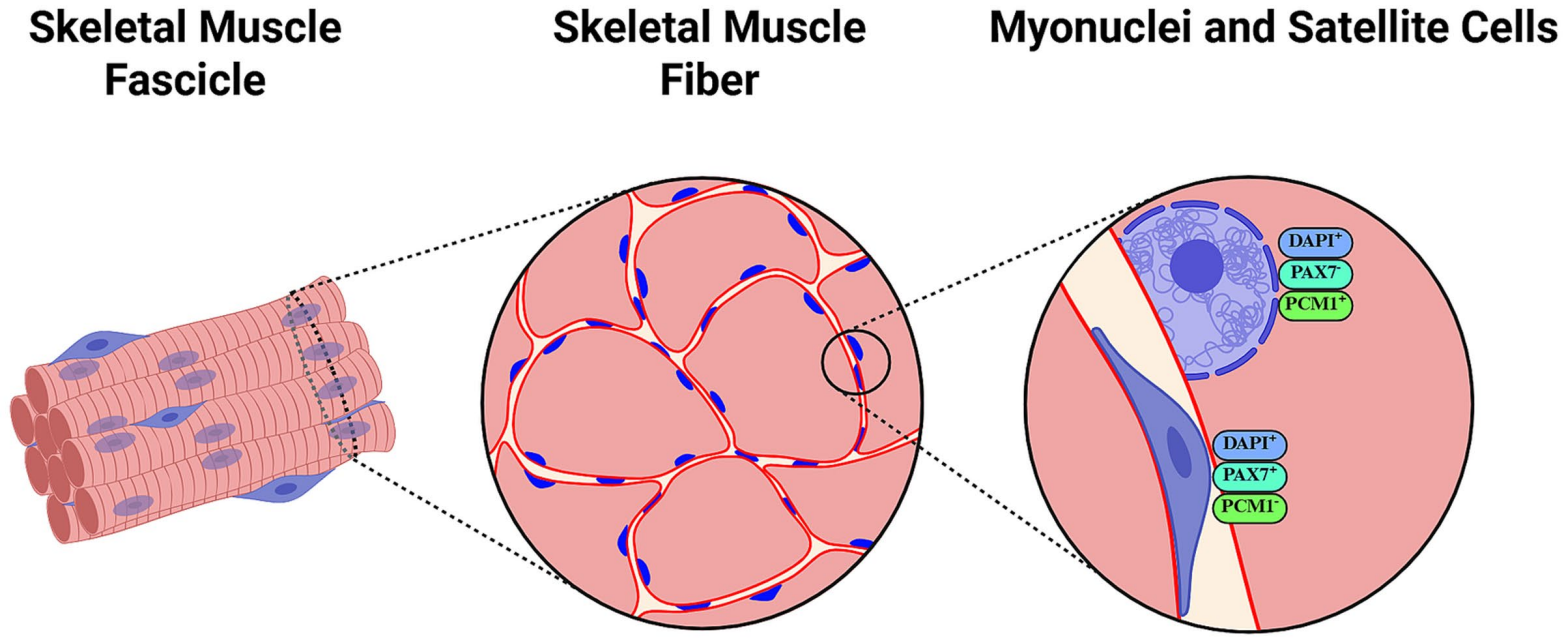
Representation of histological assessments of myonuclei and satellite cells using antibody labeling and counterstaining methods. 4′,6-diamidino-2-phenylindole (DAPI) is a popular fluorescent DNA stain frequently used in combination with antibodies against dystrophin/laminin to differentiate fiber limits. Laminin immunostaining labels the basal lamina and is particularly useful for localizing satellite cells (168), while dystrophin staining outlines the sarcolemma and can aid in distinguishing myonuclei inside the inner rim of the dystrophin ring (74). Paired Box 7 (Pax7) is typically assessed as a reliable reporter of activated, proliferating satellite cells. Pericentriolar Material-1 (PCM1) has been suggested as a specific marker of myonuclei that may help differentiate these from satellite cell nuclei. Created with BioRender.com.

Findings discussed above are dissonant with many previous (76–78), contemporaneous (79, 80) and later (8, 81–85), experiments conducted in rodents in different situations involving muscle mass loss. Numerous hypotheses have been proposed to explain dissimilarities between studies. An important one argues that Bruusgaard et al. counted myonuclei within the inner rim of the dystrophin ring, whereas most previous research may have counted satellite, stromal or immune cells as myonuclei, which may be more susceptible to apoptosis (74). However, some studies labeling the cell border (e.g., using antibodies against dystrophin and laminin), myonuclei [e.g., DNA labeling using 4′,6-Diamidino-2-Phenylindole (DAPI)], and removing satellite cells (Pax7^+^/DAPI^+^ cells) from analyses have reported myonuclei loss after detraining (8) and hindlimb suspension (84). More novel methodologies including labeling with antibodies against pericentriolar material 1 (PCM1) protein have been proposed to specifically identify myonuclei (86), yet such specificity has been questioned by some authors (87) (Figure 2).

Different methodological issues discussed in literature consist of the use of isolated single fibers versus *in vivo* nuclear labeling using HSA-rtTA; TRE-H2B-GFP (HSA-GFP) mice—a transgenic research model in which the human skeletal actin promoter drives reverse tetracycline transactivator (rtTA) expression in muscle fibers, enabling doxycycline-inducible nuclear GFP labeling without the need for immunostaining (88, 89). Fiber and muscle type might also be relevant factor to be considered. As mentioned before, type I oxidative fibers—fibers expressing myosin heavy chain I (MyHC I)—typically contain more myonuclei with consequently smaller myonuclear domains than glycolytic fibers (MyHC II), which is justified based on their greater mRNA transcriptional demand (90). For instance, Murach et al. reported that adult mice muscle fibers in *plantaris* and *gastrocnemius* lose training-induced adaptations including myonuclear content following detraining from PoWeR; however, *soleus* muscle remained unresponsive to detraining with type I oxidative fibers having elevated myonuclear number in absence of hypertrophy (91). The degree of CSA loss and the atrophy model (e.g., synergistic ablation, denervation, detraining, etc.) might also contribute to the heterogeneity of previous findings. While Bruusgaard et al. reported maintained myonuclear content in *soleus* muscle following 2 weeks of suspension despite 34% decreased myofiber CSA, different authors reported a 27% decreased myonuclei count with 54% decreased CSA under similar conditions (80). In addition, novel hypertrophy protocols in rodent studies are proposed to better represent physiological hypertrophy in humans. Synergistic ablation and denervation models, which apply continuous and supraphysiological stress to studied muscles, involve extensive rupture and regeneration processes, thus potentially act as confounding factors. In this sense, studies using PoWeR have documented decreased myonuclear content after detraining in, at least, some muscles (8, 91), whereas more recent studies using ladder climbing exercise have reported maintained myonuclei number after 10 weeks of detraining in 4–9 week-old rats (20). The age of tested animals may be relevant given the mentioned differential roles of satellite cells in muscle accretion during developmental and fully growth adult stages of life (8). This is clearly relevant when comparing rats and mice, animals with different developmental milestones. Curiously, experiments conducted in simpler models such as the tobacco hawkmoth, whose intersegmental muscles lack regenerative capacity and do not contain other cells that might confound myonuclei observations, have revealed that 75–80% muscle mass and CSA reduction leads to no change in nuclear number or DNA content (92). A summary of preclinical studies evaluating changes in myonuclear content in response to muscle unloading/atrophy in rodents, using staining techniques to delineate fiber borders and nuclei is presented in Table 1.

**Table 1:** Preclinical studies evaluating myonuclear content in response to muscle unloading and atrophy.

Authors and year of publication	Rodent model	Unloading/atrophy model	Biopsied muscle	Staining methods	Authors’ conclusions on myonuclear content
Dupont-Versteegden et al., 1999 (170)	Adult female Sprague–Dawley rats	*Unloading/atrophy*: *S*pinal cord transection, 5–10 d *Reloading*: Cycling exercise, 5 d	*Soleus*	Nuclear staining (Hoechst 33258) with cell border identification (anti-dystrophin Ab and anti-laminin Ab)	Significant decrease in myonuclei number with spinal cord transection. No change in myonuclear number with exercise
Dupont-Versteegden et al., 2000 (171)	Adult female Sprague–Dawley rats	*Unloading/atrophy*: Spinal cord transection, 4–8 wk *Reloading*: Cycling exercise with or without fetal spinal cord transplantation, 4 wk	*Soleus & Plantaris*	Nuclear staining (Hoechst 33258) with cell border identification (anti-dystrophin Ab)	No change in myonuclear number on plantaris muscle. In soleus, myonuclear number significantly decreased with spinal cord transection, and recovered with the combination of exercise and transplantation
Leeuwenburgh et al., 2005 (172)	6-mo- and 32-mo-old male Fischer 344 X Brown Norway rats	*Unloading/atrophy*: Hindlimb suspension, 2 wk	*Soleus*	Nuclear staining (Hoechst 33258) with cell border identification (anti-dystrophin Ab)	Hindlimb suspension caused myonuclear loss in young, but not in old rats
Bruusgaard et al., 2008 (72)	Female NMRI/TIE2-GF mice *(age not reported)*	*Unloading/atrophy*: Denervation (21 d), nerve impulse blockage (21 d), or mechanical unloading (tenotomy of antagonist muscles, 14 d)	*EDL & Soleus*	*In-vivo* time lapse and nuclear staining (Hoechst 33342) with cell border identification (anti-dystrophin Ab)	No significant loss of myonuclei in response to atrophy
Matsuba et al., 2009 (173)	8-week-old male C57BL/6J mice	*Unloading/atrophy*: Hindlimb suspension, 8 wk	*Soleus*	Nuclear staining (DAPI), cell border identification (anti-laminin Ab) and satellite cell specific marker (anti-Pax7 Ab)	No significant change in myonuclei numbers with hindlimb suspension unloading
Bruusgaard et al., 2010 (73)	Female NMRI mice and male Wistar rats *(age not reported)*	*Loading/hypertrophy*: Synergistic ablation, 2–3 wk *Unloading/atrophy*: Denervation, 3 mo	*EDL*	Nuclear staining (Hoechst) with cell border identification (anti-dystrophin Ab)	A significant increase in myonuclei number with loading was shown, which did not decrease during unloading at any time point
Bruusgaard et al., 2012 (74)	12-wk-old female Wistar rats	*Unloading/atrophy*: Hindlimb suspension, 2 wk *Reloading*: normal activity, 2 wk	*Soleus*	Nuclear staining (Hoechst 33342) with cell border identification (anti-dystrophin Ab)	No significant loss of myonuclei with unloading and no recruitment of myonuclei with reloading
Jackson et al., 2012 (75)	4-5-mo-old female Pax7-DTA mice	*Unloading/atrophy*: Hindlimb suspension, 2 wk *Reloading*: Normal activity, 2 wk	*Soleus & Gastrocnemius*	Nuclear staining (DAPI), cell border identification (Anti-dystrophin Ab) and satellite cell specific marker (anti-Pax7 Ab)	No change in myonuclear numbers after hindlimb suspension, with or without reloading
Sandona et al., 2012 (79)	Male C57BL/10J mice *(age not reported)*	*Unloading/atrophy*: microgravity (*s*paceflight), 20 d	*Soleus & EDL*	Nuclear staining (DAPI) with cell border identification (anti-laminin Ab)	No significant change in myonuclei number. Trend toward reduced myonuclei content in soleus
Egner et al., 2013 (19)	Adult female NMRI mice	*Loading/hypertrophy*: Testosterone treatment with or without synergist ablation, 2 wk *Unloading/atrophy*: treatment removal, 3 wk *Reloading*: Re-exposure to muscle overload, 6 d	*Soleus & EDL*	Nuclear staining (Hoechst 33342) with cell border identification (anti-dystrophin Ab)	Anabolic steroid exposure induced an increase in myonuclear number that persists beyond the cessation of treatment. Myonuclei acquired remained elevated in both soleus and EDL muscles for at least 3 mo post-treatment, despite complete reversal of muscle hypertrophy. This persistent elevation in myonuclear content significantly enhanced the muscles’ capacity for regrowth upon re-exposure to overload
Itoh et al., 2014 (80)	10-wk-old male ICR mice	*Loading/hypertrophy*: Stand-up exercise, 7 d *Unloading/atrophy*: Tail suspension, 2 wk *Reloading*: Stand-up exercise, 7 d	*Soleus*	Nuclear staining (DAPI), cell border identification (anti-dystrophin Ab) and satellite cell specific marker (anti-Pax7 Ab)	A significant decrease in myonuclei number was shown with unloading, which was recovered during reloading
Nakanishi et al., 2016 (82)	12-wk-old female Wistar rats	*Unloading/atrophy*: Hindlimb suspension, 2 wk *Reloading*: Unrestricted movement, 5 d	*Soleus*	Nuclear staining (DAPI), cell border identification (anti-dystrophin Ab) and satellite cell specific marker (anti-Pax7 Ab)	A significant decrease in myonuclei number was shown with hindlimb suspension, which was only partially recovered with reloading
Lee et al., 2018 (9)	Female Sprague–Dawley rats (8-week-old at pretraining)	*Loading/hypertrophy*: Weight-loaded ladder climbing exercise, for 8 wk *Unloading/atrophy*: No training for 20 wk *Reloading*: Weight Weight-loaded ladder climbing exercise, for 8 wk	*FHL & TA*	Nuclear staining (DAPI), cell border identification (anti-laminin Ab) and satellite cell specific marker (anti-Pax7 Ab)	The significant increase in myonuclei number with loading was not lost during detraining, and accrued myonuclei assisted with muscle hypertrophy after subsequent retraining
Dungan et al., 2019 (8)	≥4-mo-old female C57BL/6J mice	*Loading/hypertrophy*: Progressive weighted-wheel-running (PoWeR), for 8 wk *Unloading/atrophy*: Detraining, 12 wk	*Plantaris*	Nuclear staining (DAPI), cell border identification (anti-dystrophin Ab and anti-laminin Ab) and satellite cell specific marker (anti-Pax7 Ab)	An increase in myonuclei number with loading was shown, which then decreased to pre-training values with detraining
Kneppers et al., 2019 (83)	13-wk-old male C57/B16 mice	*Unloading/atrophy*: Hindlimb suspension, 2 wk *Reloading*: Unrestricted movement, for 1–8 d	*Gastrocnemius*	Nuclear staining (DAPI), cell border identification (anti-laminin Ab) and satellite cell specific marker (anti-Pax7 Ab)	Significantly reduced myonuclei number with hindlimb suspension atrophy, which was significantly recovered from >3 days of reloading
Murach et al., 2020 (91)	4-mo-old female C57BL/6J mice	*Loading/hypertrophy*: PoWeR, 2 mo *Unloading/atrophy*: Detraining, 6 mo	*Soleus, Gastrocnemius & Plantaris*	Nuclear staining (DAPI) with cell border identification (anti-dystrophin Ab)	An increase in myonuclei number with PoWeR was shown, which was retained after detraining in soleus but not in gastrocnemius and plantaris muscle
Eftestøl et al., 2022 (20)	4-week-old male Sprague–Dawley rats	*Loading/hypertrophy*: Climbing exercise, for 5 wk *Unloading/atrophy*: Detraining, 10 wk *Reloading*: Climbing exercise, 2 wk	*TA & Soleus*	Nuclear staining (DAPI), cell border identification (Anti-dystrophin Ab) and anti-PCM1 Ab, considered as a myonucleus-specific marker	A significant increase in myonuclei number with loading was evident when compared to control. Differences were retained after detraining

Ab, antibodies; d, days; DAPI, 4′,6-diamidino-2-phenylindole; DTA, Diphtheria Toxin A; EDL, extensor digitorum longus; FHL, flexor hallucis longus; ICR, Institute of Cancer Research; mo, months; NMRI, Naval Medical Research Institute; PCM1, pericentriolar material 1; PoWeR, progressive weighted-wheel-running; SC, satellite cells; TA, tibialis anterior; wk, weeks.

Naturally, findings obtained in pre-clinical research cannot be directly translated to humans. Early studies using classic staining methods in single fibers showed that myonuclei number remained unchanged after 4 months of bed rest in middle-aged men (93). One month of recovery was sufficient to induce hypercompensation in CSA and myonuclear content values compared to baseline, which author attributed to increased physical activity during post-bed-rest ambulation (93). Later clinical studies reported no change in myonuclear content of middle-aged men after 28 days bed rest (94), and in young men after 2 weeks of knee full leg cast immobilization (95). On the other hand, subtle decrements (5%) in myonuclear content in type I and II fibers were reported in a cohort of seven middle-aged adults (49–54 year olds) after 15 days bed rest (96). Studies exploring more moderate models of episodic disuse have documented unchanged myonuclear content in both young and older adults subjected to 5 days one-legged knee immobilization (97, 98), and to 5–7 days bed rest (99, 100), despite significant decrements in assessed muscle CSA, as well as after 14 days step reduction in older adults (101, 102). Based on these findings, mild episodic disuse does not appear to impact myonuclei number compared to baseline values in humans independently of age, while limited evidence suggests that more prologued disuse protocols might have a subtle impact in middle-aged adults.

Whether myonuclei gained during training exercise can be lost after subsequent detraining, or faster regained during retraining has not been explored until recently. Blocquiaux et al. conducted a prospective exercise intervention trial in 40 healthy older men, aged 58–70 years old, using a training-detraining-retraining approach (103). The protocol consisted of 12 weeks of whole-body resistance training sessions of 60 min, three times per week. Following this training period, subjects were requested to return to normal physical activity habits for 12 weeks followed by another 12 weeks of retraining under similar conditions. Muscle biopsies were collected from vastus lateralis muscle, and immunostaining was performed to differentiate satellite cells and myonuclei using Pax7^+^, laminin, and PCM1 antibodies. Although methods were sound, the protocol failed to elicit significant changes in CSA and myonuclear number, thus compromising the evaluation of muscle memory during detraining and retraining steps. Nonetheless, detraining was linked to significantly decreased number of myonuclei in type I fibers and a trend in fiber II compared to peak values, while retraining led to higher myonuclear number per type II fiber (103). In a later study, Psilander et al. recruited 19 healthy inactive volunteers aged ≈25 years old who were subject to two strength-based training periods separated by 20 weeks of detraining (104). The first period consisted of 10 weeks of unilateral training while the second included bilateral training for 5 weeks only (104). Muscle biopsies were collected from *vastus lateralis* muscle and myofiber cryosections were processed for myonuclei visualization (104). While CSA gains were achieved during training, no decrement in CSA was documented during detraining, and neither training nor detraining impacted myonuclei number (104). A re-analysis of their raw data conducted by Murach et al. focusing only on subjects who gained myonuclei during training suggested that these may be lost after detraining in these individuals (105), yet this statistical approach can be subject to bias since selecting a different subset of individuals may lead to dissimilar interpretations of myonuclei content during detraining (106).

More recent clinical studies have shed light upon this subject. Nielsen et al. were first to describe a persisting anabolic effect of former usage of AAS in muscle hypertrophy, extending to humans findings from previous pre-clinical research from Bruusgaard and Gundersen’s lab (24). They conducted a cross-sectional analysis of 25 participants including eight current AAS users, seven former users, and 10 controls with no history of usage (24). A key observation consisted of higher myonuclear density and DNA to plasma ratio in former AAS users compared to control in type II fibers, and a trend in mixed fibers (24). No difference in CSA was observed for any fiber type (24). In a recent short report, six patients of unilateral Achilles tendon rupture (27–63 years old) who underwent standard suture repair procedures were recruited, and biopsies were taken from *gastrocnemius* muscles 2–5 days after the injury and after ≈6 weeks of immobilization for myofiber cross-section analyses through DAPI-PCM1-dystrophin antibody counterstaining (22). Interestingly, myonuclei content did not change in type I fibers, and even tended to increase in type II of the injured muscles (22). Nonetheless, it should be mentioned that this study consisted of a relatively small analysis with high interindividual variability in the immobilization response, and it remains to be clarified whether these findings can be translated to longer immobilization periods in wider populations. Last, a following study conducted by Cumming et al. (21) aimed to address some methodological concerns of their previous trial (104) using a similar training-detraining-retraining protocol in young healthy subjects (21). In this case, subjects participated in a 10-week unilateral elbow flexor resistance training, followed by 16 weeks detraining, and another 10 weeks of retraining were both arms were exercised. Since elbow flexor is not involved in weight bearing activities to the same extent as *vastus lateralis*, authors expected to better isolate the effects of detraining (21). Results from this study revealed that myonuclei number increased in both fiber types after the first training period, no change was observed during detraining in terms of myonuclear density, and a further increase was reported after the retraining period (21), which is supportive of myonuclear permanence after detraining in humans. However, these findings did not translate to enhanced hypertrophic response during re-training. Authors argued that individual participants with the highest myonuclear density also experienced the highest degree of hypertrophy during retraining, and longer detraining periods may be needed in future studies to test these findings (21). Overall, recent longitudinal clinical research appears to support a certain degree of myonuclei permanence as a characteristic of skeletal muscles following moderate long-term disuse and detraining. Whether this phenomenon may partially underlie an enhanced hypertrophy response following detraining periods reported in previous literature (107), remains to be clarified, and different mechanisms most notably epigenetic marks have been suggested to play a role. A summary of clinical studies evaluating changes in myonuclear content in response to muscle unloading/atrophy, using staining techniques to delineate fiber borders and nuclei is presented in Table 2.

**Table 2:** Clinical studies evaluating myonuclear content in response to muscle unloading and atrophy.

Authors and year of publication	Population of study	Unloading/atrophy model	Biopsied muscle	Staining methods	Authors’ conclusions on myonuclear content
Brooks et al., 2010 (94)	Healthy male adults, 31–55 y; *n* = 31	*Unloading/atrophy*: Bed rest, with or without EAA supplementation or resistance exercise sessions, 28 d *Reloading*: Active recovery (treadmill and resistance exercise), 14 d	*Vastus lateralis*	Nuclear staining (Hoechst 33342), cell border identification (anti-laminin Ab) and satellite cell specific marker (anti-Pax7 Ab)	No significant decrement in myonuclei number after muscle atrophy from bed rest was shown in any group
Dirks et al., 2014 (97)	Healthy male older adults, 69 ± 1 y; *n* = 23	*Unloading/atrophy*: One-legged knee immobilization, with or without protein supplementation, 5 d	*Vastus lateralis*	Nuclear staining (DAPI), cell border identification (anti-laminin Ab) and satellite cell specific marker (anti-CD56 Ab)	No decrease in myonuclear numbers after immobilization for both groups
Dirks et al., 2014 (98)	Healthy male young adults, 23 ± 1 y; *n* = 24	*Unloading/atrophy*: One-legged knee immobilization, with or without NMES sessions, 5 d	*Vastus lateralis*	Nuclear staining (DAPI), cell border identification (anti-laminin Ab) and satellite cell specific marker (anti-CD56 Ab)	No significant change in myonuclei number after immobilization in any group
Snijders et al., 2014 (95)	Healthy male young adults, 24 ± 1 y; *n* = 12	*Unloading/atrophy*: One-legged knee immobilization, for 2 wk *Reloading*: Natural rehabilitation, 6 wk	*Vastus lateralis*	Nuclear staining (DAPI), cell border identification (anti-laminin Ab) and satellite cell specific marker (anti-CD56 Ab)	No significant change in myonuclei numbers between baseline, post-immobilization and post-rehabilitation was reported
Arentson-Lantz et al., 2016 (96)	Healthy middle-aged adults, 51 ± 1 y; *n* = 7 (4 male, 3 female)	*Unloading/atrophy*: Bed rest, 2 wk	*Vastus lateralis*	Nuclear staining (DAPI), cell border identification (anti-laminin Ab) and satellite cell specific marker (anti-Pax7 Ab)	Myonuclei content significantly decreased after 14 days of bed rest
Dirks et al., 2016 (99)	Healthy male young adults, 23 ± 1 y; *n* = 10	*Unloading/atrophy*: Bed rest, 1 wk	*Vastus lateralis*	Nuclear staining (DAPI) and cell border identification (Anti-laminin Ab)	No significant change in myonuclei number following bed rest
Reidy et al., 2017 (100)	Healthy older adults, 60–80 y; *n* = 20 (17 male, 3 female)	*Unloading/atrophy*: Bed rest, with or without NMES and protein supplementation, 5 d	*Vastus lateralis*	Nuclear staining (DAPI), cell border identification (anti-laminin Ab) and satellite cell specific marker (anti-Pax7 Ab)	Only a trend toward decreased myonuclei number with bed rest was reported
Moore et al., 2018 (101)	Healthy male older adults, 71 ± 5 y; *n* = 14	*Unloading/atrophy*: Step count reduction, with or without concomitant lower leg unilateral resistance exercise, 14 d *Reloading*: Normal activity, 14 d	*Vastus lateralis*	Nuclear staining (DAPI), cell border identification (anti-laminin Ab) and satellite cell specific marker (anti-Pax7 Ab)	No significant changes in myonuclei numbers with step count reduction was detected
Psilander et al., 2019 (104)	Healthy young adults, 25 ± 1 y; *n* = 19 (9 male, 10 female)	*Loading/hypertrophy*: Unilateral strength training, 10 wk *Unloading/atrophy*: Detraining, 20 wk *Reloading*: Bilateral strength retraining, 5 wk	*Vastus lateralis*	Nuclear staining (DAPI) and cell border identification (anti-dystrophin Ab)	Myonuclear number remained unchanged throughout training, detraining, and retraining, indicating no significant alterations in myonuclear content across the protocol
Reidy et al., 2019 (102)	Healthy older adults, 70 ± 2 y; *n* = 12 (7 male, 5 female)	*Unloading/atrophy*: Step count reduction, 14 d	*Vastus lateralis*	Nuclear staining (DAPI), cell border identification (anti-laminin Ab) and satellite cell specific marker (anti-Pax7 Ab)	No significant change in myonuclei number per fiber across the intervention regardless of fiber type
Blocquiaux et al., 2020 (103)	Healthy male older adults, 57–77 y; *n* = 40	*Loading/hypertrophy*: Resistance exercise training, 12 wk *Unloading/atrophy*: Detraining, 12 wk *Reloading*: Resistance exercise training, 12 wk	*Vastus lateralis*	Nuclear staining (Hoechst 33342), cell border identification (anti-laminin Ab), satellite cell specific marker (anti-Pax7 Ab), and anti-PCM1 Ab, considered as a myonucleus-specific marker	No significant change in myonuclei number with training, but significant decrease in myonuclei content in type I fibers and a trend toward decreased myonuclei number in type II fibers with detraining
Nielsen et al., 2023 (24)	Healthy active adults with current (*n* = 8), prior (*n* = 7) or no (*n* = 10) AAS use, 18–50 y; *n* = 25 (25 male, 0 female)	*Loading/hypertrophy*: Resistance exercise training with AAS use, ~3 y *Unloading/atrophy*: Resistance exercise training with AAS discontinuation, ~4 y	*Vastus lateralis*	Nuclear staining (DAPI), cell border identification (anti-laminin Ab) and satellite cell specific marker (anti-Pax7 Ab)	Individuals with a history of AAS use exhibited significantly reduced myonuclear domain sizes indicative of elevated myonuclear density in type II muscle fibers compared to non-AAS-exposed controls, despite an average of 4 years having elapsed since cessation of AAS administration
Cumming et al., 2024 (21)	Healthy young adults, 24 ± 3 y; *n* = 12 (4 male, 8 female)	*Loading/hypertrophy*: Unilateral elbow-flexor strength training, 10 wk *Unloading/atrophy*: detraining, 16 wk *Reloading*: Bilateral elbow-flexor strength training, 10 wk	*Biceps brachii and brachialis*	Nuclear staining (DAPI) and cell border identification (anti-dystrophin Ab), and anti-PCM1 Ab, considered as a myonucleus-specific marker	No significant loss of myonuclei with detraining was shown while a further recruitment of myonuclei with retraining was documented
Horwath et al., 2025 (22)	Injured active adults, 43 ± 15 y; *n* = 6 (5 male, 1 female)	*Unloading/atrophy*: Lower leg immobilization following Achilles tendon rupture, 6 wk	*Gastrocnemius*	Nuclear staining (DAPI), cell border identification (anti-laminin and anti-dystrophin Ab), satellite cell specific marker (anti-Pax7 Ab), and anti-PCM1 Ab, considered as a myonucleus-specific marker	No significant loss of myonuclei with prolonged lower leg immobilization was shown

AAS, anabolic-androgenic steroids; Ab, antibodies; d, days; DAPI, 4′,6-diamidino-2-phenylindole; EAA, essential amino acids; NMES, neuromuscular electrical stimulation; PCM1, pericentriolar material 1; wk, weeks; y, years.

### 3.1 Alternative mechanisms of muscle memory

Time-course analyses of skeletal muscles following training and detraining protocols have revealed different adaptations that might be particularly resilient to the cessation of the anabolic stimuli. Examples of these may include maintained capillarization and mitochondrial enzymatic activity (22, 108), differential micro-RNA (miRNA) expression (91), and stable DNA and histone modifications acting as epigenetic regulators. Particularly, the possibility of epigenetic mechanisms of muscle memory impacting gene expression have gained great attention in recent years. For instance, Cumming et al. reported that previously trained muscle conserved differential expression of genes compared to control muscle during detraining (EGR1, MYL5, COL1A1), which were considered to be relevant for skeletal muscle performance or development (21). Further, decreased gene expression was observed after retraining in the previously trained arm compared to control (21). Interestingly, genes related to ribosomal structure and function were particularly enriched in the previously trained muscle before retraining (21). This argues in favor of a potential epigenetic memory in skeletal muscles, which may persist and provide an advantage after a further anabolic stimulus.

Both acute and repeated resistance exercise have been reported to elicit a state of general hypomethylation in CpG sites—cytosines adjacent to guanines separated by a phosphate group—of muscle tissue in humans (109, 110), which appears to be retained during prolonged periods (e.g., 7 weeks) of muscle unloading. Bioinformatic analyses conducted in public datasets have further supported these findings observing a larger proportion of hypomethylated compared to hypermethylated genes after acute and chronic resistance exercise (111). Five genes expressed after exercise were reported to remained hypomethylated after detraining following previous training stimulus (111). Also, several hypomethylated genes reported in some of these studies relate to PI3K-Akt signaling, potentially explaining a role of hypomethylation in regulating MPS (109, 110). Promoted DNA hypomethylation suggestive of epigenetic muscle memory has also been reported in young and older individuals following training-detraining-retraining protocols (112). Enriched hypomethylation of growth-related pathways was differentially observed in myonuclei compared to interstitial nuclei of a mouse model of conditional, inducible fluorescent myonuclear labeling (HSA-GFP mice) subjected to PoWeR and detraining protocols (113). Relative to untrained control mice, myonuclear hypomethylation was reported in promoter regions of genes linked to mTOR, autophagy, mitochondrial biogenesis, and other growth-related pathways (113). Notably, accelerated regain of muscle mass in previously trained mice was reported, and authors speculated that the faster hypertrophy response to retraining was caused by enduring epigenetic effects of training on myonuclear DNA methylation, miRNA modifications, and interstitial nuclei contributions, among different factors (113). In a later study by the same group, methylome differences between resident myonuclei and recently accrued myonuclei derived from satellite cells were observed after a bout of resistance exercise in mouse models (89). Authors found that resident myonuclei favored hypomethylation of genes involved in PI3K-Akt and FoxO pathways, as opposed to newly added myonuclei which favored hypomethylation of RNA polymerase II-mediated transcription, cell-to-cell adhesion pathways, and ribosomal RNA subunits (89). Based on these findings, it was hypothesized that resident versus accrued myonuclei roles after load-induced hypertrophy could differ, with resident myonuclei being directly involved in protein turnover regulation and newly accrued myonuclei taking a more supportive role, enhancing satellite cell proliferation and fusion, ribosomal biogenesis and ECM remodeling (89).

Collectively, current evidence suggests that skeletal muscle hypertrophy may be linked not only to the accretion of myonuclei which might persist following periods of atrophy, but also to myonuclear epigenetic signatures that may support enhanced adaptation during retraining. Nonetheless, further research is needed to clarify the nature and consequences of epigenetic changes induced by training and detraining. For example, recent research indicated evidence of epigenetic muscle memory following endurance exercise (HIIT), albeit no enhancement in physiological variables response [e.g., maximal oxygen consumption (VO_2_ max)] was documented after retraining, and the consequences of these changes remain to be investigated (114). Additionally, a recent study conducted in middle-aged women and men identified signatures of proteomic memory after detraining and retraining protocols. Further comparisons with previously cited epigenetic studies revealed carbonyl reductase 1 (CBR1) and calpain-2 as resistance training-induced “proteomic memory proteins” (115). These findings warrant further investigation to better understand their implications for long-term muscle adaptation.

## 4 Implications of muscle memory and research opportunities

### 4.1 Long-term detraining periods

The potential implications of skeletal muscle memory in sports have primarily been discussed in the context of enhanced hypertrophic responses in former AAS users. This is based on the hypothesis that the peak myonuclear density achieved during AAS use, coinciding with the muscle fiber’s largest historical size, results in a sustained increase in myonuclear number and possibly epigenetic modifications, both of which may facilitate a heightened hypertrophic response years after AAS discontinuation (23). It should be noted that powerlifters, as a frequent example of AAS-abusers, are not typically expected to detrain, and evidence on myonuclear permanence in settings of detraining might not necessarily reflect on the situation that occurs during continuous training (23).

From a more positive standpoint, the potential implications of enhanced hypertrophic response in mitigating muscle mass loss during periods of detraining have received little attention. Detraining is the partial or complete loss of loading-induced adaptations in response to an insufficient training stimulus (116). Short-term detraining typically consists of periods <4 weeks of training cessation, while longer periods are considered long-term detraining (116, 117). Short-term detraining occurs after cessation of structured training exercise, where skeletal muscle usually witnesses a reduction in fiber CSA and total muscle mass, as well as a moderate decrease in maximal dynamic strength (116). Long-term effects are more pronounced and may lead to a decline in muscle fiber CSA to pretraining levels, as well as a gradual shift in fiber populations, with proportions of oxidative fibers seeing a decrease in endurance athletes and an increase in strength-trained athletes (117). Strength performance, isokinetic strength and force production also decline significantly through longer periods of detraining, with resistance-trained athletes seeing the largest decline (117). Evidence on myonuclear permanence during a 16 week-detraining period might support a role of resident myonuclei in facilitating muscle regain during retraining (21), albeit findings from preclinical models in terms of improved retraining capabilities (104) have not been clearly replicated in humans (21).

Probably, the most relevant case of long-term detraining in sports settings consists of recovery from injury requiring immobilization (22). Findings from the previously cited study conducted by Horwath et al. indicate evidence of myonuclear permanence in *gastrocnemius* muscle fibers after 6 weeks of immobilization due to recovery from Achilles tendon rupture (22). Increased myonuclear density might be speculated to facilitate muscle regain in the immobilized muscles and may be beneficial for accelerating the return to competition, which remains to be confirmed. Additional research opportunities may include clarifying how variations in exercise type, volume and intensity influence myonuclear accretion during hypertrophy, with the goal of developing preventive strategies that support muscle regain following extended periods of detraining in athletes. In addition, it remains to be explored whether preventive exercise programs can be tailored to induce long-lasting adaptations in specific muscle groups that are particularly vulnerable to sport-related injuries. On the other hand, emerging evidence suggests that injury might also associate with a “negative” epigenetic memory that may act as an effector of the loss of muscle mass and plasticity linked to poor functional recovery after surgery (118). How epigenetics may contribute to functionality recovery and the role that repetitive injuries may play have not been explored to date. Further research leveraging training-detraining-retraining protocols might expand our knowledge on different mechanisms implicated in muscle memory and shed light on these questions. These studies would benefit from including athletes of different sexes, particularly given the dearth of research in female populations.

### 4.2 Age-related muscle wasting

As mentioned, aging is associated with accelerated loss of muscle mass and quality, known as sarcopenia, which may predominantly affect type II fibers (119). This atrophy has been linked to a reduction in satellite cell content, which may also impact type II fibers to a greater extent (120, 121), and a diminished capacity for satellite cell recruitment following resistance exercise (122, 123). Studies have shown that satellite cell number correlates with fiber size in aged muscle (124), and impaired recruitment may contribute to reduced myonuclear accretion. For instance, a study conducted by Karlsen et al. reported a ≈ 50% reduction in satellite cell content per type II fiber in individuals aged 83–94 years old, along with smaller myonuclear domains and a trend toward lower myonuclear content compared to young adults (125). Notably, a 12-week resistance training program failed to induce significant type II fiber hypertrophy in these older adults, which was linked to blunted satellite cell response, underscoring the importance of satellite cell activity in muscle adaptation in long-lived individuals (125). While these findings are not entirely consistent across all studies (126, 127), methodological differences and age disparities (e.g., <70 vs. 80–90 years old) may explain these discrepancies (125).

**Figure 3:**
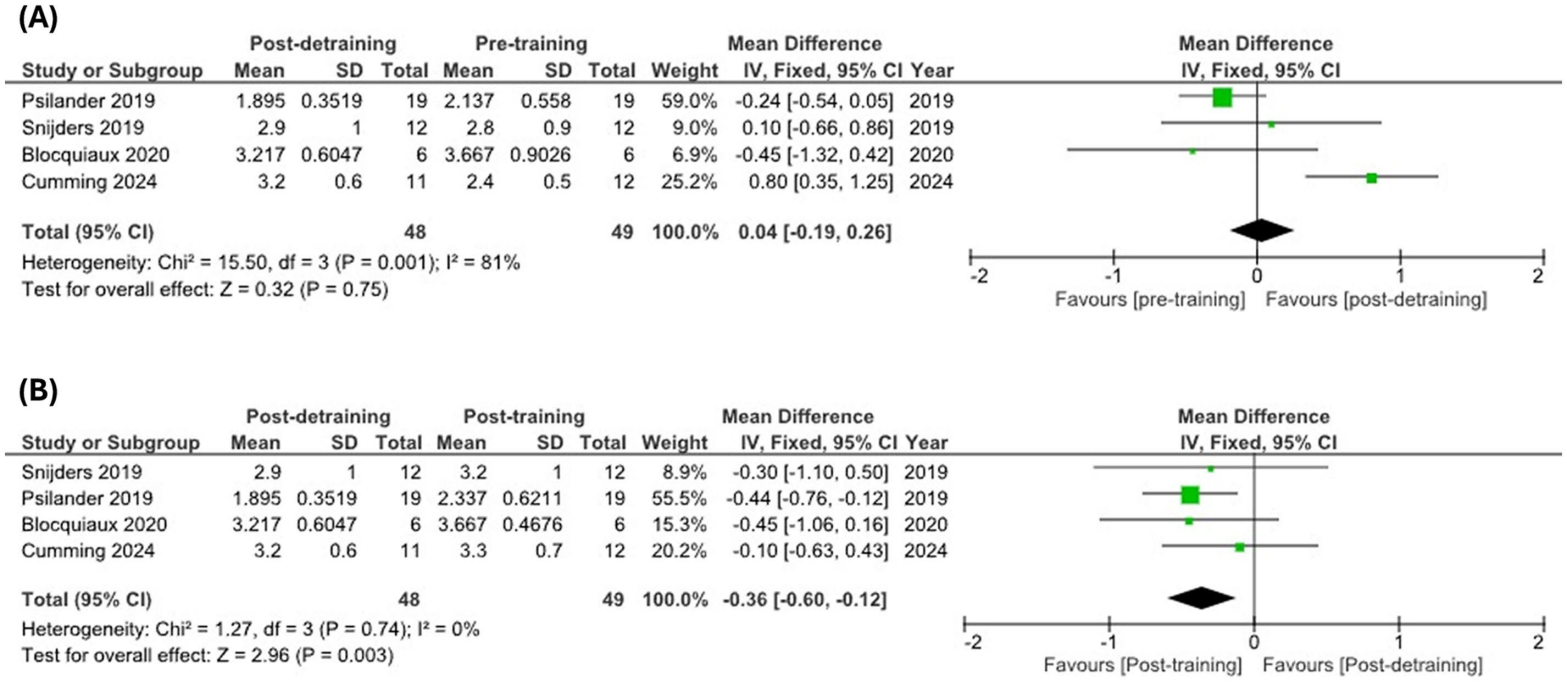
Meta-analysis of published data from longitudinal studies examining myonuclei content in type II fibers during training-detraining protocols. Myonuclear content pre-training versus post-detraining **(A)** and post-training versus post-detraining **(B)** in type II fibers. Meta-analysis was conducted using RevMan v5.4.

Several cross-sectional analyses using immunostaining techniques to estimate myonuclear number excluding satellite cell counts have documented maintained myonuclei content in type II fibers in older compared to young individuals (20s vs. 60s years old) (128, 129), while others have consistently reported decreased myonuclear content in type II fibers of older individuals over 70 years old (121, 125, 130, 131). This suggests that myonuclei loss may characterize type II fiber atrophy in aged skeletal muscles, which might be more evident in long-lived subjects (i.e., >70 years old). In alignment, all available longitudinal analyses showing either a trend or a significant loss of myonuclei in type II fibers following detraining have been conducted in older adults (103, 132), while those reporting maintained myonuclei content after detraining recruited younger individuals (≈25 years old) (21, 104). However, despite discrepancies in single studies recruiting individuals of different age, a recent meta-analysis including three of these records concluded that myonuclear content significantly decreases after a detraining period to numbers below pre-training values without significant differences being observed between young and older adults (133). These conclusions are not aligned with findings of maintained myonuclei content after moderate disuse, and detailed inspection of the analyses conducted reveals some methodological considerations. Authors of the meta-analysis reported post-detraining and pre-training comparisons in order to assess whether myonuclei number decreases below baseline values after detraining, which would clearly confirm myonuclei loss (133). It was stated that pooled estimates of mean differences and 95% confidence intervals (95% CI) were calculated, yet analyses of myonuclear content in type II fibers after detraining [Figure 2SL from Rahmati et al. (133)] directly included mean ± standard deviation (SD) data from some pooled studies (132). Also, authors included data reported as 95% CI by Psilander et al. (104), but these were stated to be normalized to the first biopsy from the respective leg in the original study, leading to narrower confidence intervals in the pooled analysis. Consequently, Psilander’s research contributes to >94% of the weight in the meta-analysis, favoring pre-training myonuclear content compared to post-detraining values. Fortunately, Blocquiaux et al. (103) and Psilander et al. (104) also reported raw data from their studies, which enables the opportunity to re-analyze these findings. Using these data, we conducted a re-analysis to explore changes in myonuclear content in type II fibers following detraining [including new data reported by Cumming et al. (21)] (Figure 3). According to our re-analysis, myonuclei content in type II fibers, in fact, does not appear to decrease below pre-training values after detraining (Figure 3A), but neither does current evidence support peak myonuclei content being completely retained after a prolonged detraining period (Figure 3B). These data may point to a delayed myonuclear removal process relative to other cellular components, rather than supporting the notion of indefinite permanence. Naturally, these analyses include a very limited number of studies, and further research is needed to strengthen these findings and clearly evaluate age-based differences.

In summary, different reports have indicated reduced satellite cell content and activity in older type II myofibers, which may underlie decreased nuclei number and potentially contribute to type II fiber-specific atrophy. Limited evidence would be supportive of delayed removal of myonuclei compared to different cellular components, rather than indefinitely preserved myonuclear content throughout life. Mechanisms involved in a potential age-related decline in myonuclei content remain unclear, but authors have speculated that genomic instability resulting from cumulative DNA damage might pose a challenge to postmitotic cells such as myofibers lacking cellular turnover mechanisms (134).

Promoting satellite cell activity and proliferation through exercise and targeted nutritional strategies may be a logical approach to facilitate myonuclear accretion and potentially mitigate age-related skeletal muscle wasting. Furthermore, emerging evidence suggests that satellite cells might contribute to muscle maintenance and adaptation through non-fusion mediated mechanisms, albeit most evidence emerges from preclinical models (135). As mentioned before, these effects might include remodeling of the ECM through secretion of factors such as metalloproteinases (136) and miRNA-containing extracellular vesicles involved in epigenetic regulation (137), but also potentially preserved capillarization (135) and maintained neuromuscular junction (138). Satellite cells may also mediate interactions between macrophages and fibrogenic cells during mechanical loading (139), further highlighting their multifaceted role in muscle adaptation. Notably, recent studies have reported a greater number of satellite cells associated with type II myofibers in lifelong recreationally active older adults compared to their sedentary counterparts (140). This suggests that, beyond increasing myonuclear content, a sustained satellite cell pool may be a beneficial outcome of lifelong physical activity, with important implications for muscle growth and preservation. Naturally, these broader roles of satellite cells may also be relevant in contexts of disuse and prolonged detraining previously discussed.

### 4.3 Nutritional strategies

Nutritional strategies leveraged by athletes aiming at consistently promoting MPS and subsequent hypertrophy in the context of regular resistance exercise, when maintained over time, may lead to sustained myonuclear accretion through promoting satellite cell responses (23). As discussed in previous sections, maintained myonuclear populations and transcriptional output, potentially coupled with epigenetic changes and different benefits arising from potential fusion-independent roles of satellite cells (135), may associate with increased regain of muscle mass, which might play an important role after situations of long-term detraining, such as recovery from injury. Further, increased satellite cell activity and proliferation might be beneficial for aged individuals subject to skeletal muscle atrophy to promote muscle regeneration, and, potentially, enhance muscle mass gains when coupled with resistance training.

Protein and amino acid intake promote MPS through IGF-1-Akt-mTORC1 signaling. However, whether protein/amino acid effects of MPS during robust hypertrophy may be partially mediated through promoted satellite cell activation and proliferation, and resulting fusion-mediated myonuclei accretion is unclear. In this regard, some studies have explored satellite cell responses to combinations of acute exercise and protein supplementation. Promoted expression of myogenic cell cycle-regulating genes and increased muscle DNA content was observed within 2–6 h after acute resistance exercise in young untrained men, which was independent of previous ingestion of protein (25 g) versus isoenergetic carbohydrate or non-caloric placebo (141). On the other hand, a study analyzing the effects of an acute high-intensity eccentric exercise bout in young participants showed that post-exercise intake of 28 g of whey protein associated with increased satellite cell content, particularly in type II fibers, for up to 48 h compared with baseline, which was not seen with placebo (142). Interesting research reported by Reidy et al. indicated that older adults (≈72 years old) were uncapable of achieving satellite cell activation (Pax7^+^ count) 24 h after leg resistance exercise, while a trend toward increased satellite cell activation, proliferation (Ki67^+^ and MyoD^+^ cells), and increased myonuclei content was only observed with EAA supplementation (10 g, 1 h post-exercise) (143). It was therefore concluded that satellite cell proliferative capacity is compromised in older men and can be partially rescued with postexercise EAA ingestion (143). Dissonant with these findings, severe protein restriction (0.1 g/ kg bw/ day for 4 days) was found not to significantly impact satellite cell responses to exercise compared to sufficient intakes (1.2 g/ kg bw/ day) in healthy young men (144). To our knowledge, this is the only piece of evidence on the impact of acute severe protein restriction on satellite cell activation, and further studies are needed to better understand this topic.

Other studies have analyzed the long-term effects of dietary protein intake coupled with exercise. Olsen et al. compared 20 g/day milk protein hydroxylate supplementation versus carbohydrate (considered as placebo) in combination with strength-focused resistance training for 16 weeks (26). Results indicated significantly larger gains in the relative number of satellite cells in the protein-supplemented group compared with the placebo group, but only at 4 weeks within the training program (26). Also, no changes in myonuclei counts was observed between these groups (26). In a study conducted by Farup et al., young male adults participated in a 12-week unilateral resistance training program to investigate the effects of concentric versus eccentric exercise on satellite cell activity and myonuclei number. Participants received daily supplementation on training days with either 19.5 g of whey protein combined with 19.5 g of carbohydrates or 39 g of carbohydrates alone (glucose) (25). Interestingly, results showed increased satellite cell content in type I fibers with protein supplementation, and an increase in type II fiber CSA and myonuclear accretion (myonuclear domain was not reported) when subjects performed concentric compared to eccentric resistance exercise in combination with protein supplementation (25). These findings are surprising as eccentric exercise was speculated to elicit a more potent satellite cell response due to the associated muscle damage; however, it was concentric exercise the stronger promoter of satellite cell response in all fiber types. Different reports have indicated promoted satellite cell counts with whey-based protein compared to soy protein after a 12-week training program in young male adults (145). On the other hand, Reidy et al. set off to study whether traditional resistance training with concurrent concentric and eccentric contraction may facilitate fiber-type-specific satellite cell proliferation and myonuclei addition (146). Authors comprehensively analyzed the impact of 22 g/day whey or soy protein supplementation compared to placebo during a 12-week training program (146), and concluded that protein supplementation does not further increase muscle CSA, strength, satellite cell content and myonuclear accretion on *vastus lateralis* muscle in comparison to resistance training alone (146). Last, in a 24-week randomized, double-blind, placebo-controlled trial, 34 frail elderly participants underwent resistance training with either protein (2 × 15 g/day) or placebo supplementation (147). Muscle biopsies were taken at baseline, 12, and 24 weeks to assess fiber CSA, satellite cell content, and myonuclear characteristics (147). Protein supplementation significantly enhanced both type I and II muscle fiber hypertrophy; however, no changes were observed in satellite cell or myonuclear content (147). Hypertrophy was associated with increased myonuclear domain size, suggesting growth occurred without SC activation or myonuclear accretion (147).

Based on these studies, it is complicated to elucidate the role of protein and amino acid intake in satellite cell activation and myonuclear accretion. Particularly, baseline protein intake might be sufficient to promote muscle adaptation in these trials [e.g., ≈1.3 g/kg/d in Reidy et al. research (146)], and isolating an impact of satellite cells on promoting hypertrophy in absence of damage seems challenging based on these methodologies. Likewise, additional variability may arise from differences in sociodemographic factors (sex, age) as well as methodological discrepancies (immunostaining, training program, timing of the biopsies, etc.). Pre-clinical evidence might help expanding on these mechanisms. For instance, combinations of resistance exercise (ladder climbing) and regular leucine administration associated with increased myonuclei number by promoting satellite cell differentiation in rats, which was linked to upregulated expression of IGF-1 (148). Similarly, the leucine derivate β-hydroxy-β-methylbutyrate (HMB) has been reported to promote satellite cell proliferation in fast switch muscles of aged rats, which, when combined with its anticatabolic effects, might explain observed higher muscle mass regain after reloading from immobilization (149). Altogether, while mixed findings have been documented in clinical research, promoted satellite cell response and myonuclear accretion might be a potential additional mechanism underlying skeletal muscle hypertrophy following protein/amino acid intake in combination with physical exercise, which requires deeper investigation. Permanence or temporal retention of these myonuclei might be beneficial to facilitate muscle regain after disuse or with aging, which further supports established recommendations of elevated protein intake in athletes and aged individuals compared to the general, sedentary population.

Besides protein amino acids, limited evidence supports a role of other nutritional compounds commonly associated with athletes’ diets and ergogenic supplements in promoting myonuclei accretion. A notable example is creatine monohydrate, whose supplementation (6–24 g/day), in combination with resistance exercise training, was shown to significantly increase satellite cell populations (4–6 weeks) and myonuclear accretion (4 week) compared to training with control and protein diet only (26). Mechanisms involved are not clear, but previous preclinical research speculated that simply promoting osmotic pressure in myofibers due to increased creatine content might “indirectly signal satellite cell proliferation,” which may require further clarity (150). Polyphenol ingredients often used in recovery products may contribute to satellite cell activation and proliferation. Particularly, resveratrol supplementation (500 mg/day) has been reported to increase fiber size and total nuclei content (satellite cell and myonuclei) on *vastus lateralis* muscle of older healthy men participating in a 12-week resistance and aerobic exercise program compared to placebo (151). Authors proposed that these effects might be mediated by enhanced satellite cell proliferation but also speculated that suppression of pro-apoptotic mechanisms might be involved (151). Previous preclinical research from the same group documented increased myogenic proliferation and decreased apoptotic signaling in aged rats administered green tea extract after reloading following muscle disuse (14 days of hindlimb suspension) (152). In a study involving mice subjected to a 7-day hindlimb immobilization followed by a 7-day recovery period, treatment with either curcumin or resveratrol via peritoneal injection led to an increase in progenitor, activated, quiescent, and total muscle satellite cells compared to placebo, indicating enhanced muscle regenerative potential post-immobilization (153). However, mixed results of polyphenol supplementation and satellite cell response in humans have also been reported in literature (154).

Interestingly, polyphenols and polyphenol-rich ingredients such as resveratrol, curcumin and green tea catechins are demonstrated to activate Sirtuin 1 (SIRT1) (155–157), a NAD^+^-dependent histone deacetylase involved in satellite cell proliferation and myonuclear accretion (158, 159). SIRT1 deacetylase activity promotes peroxisome proliferator-activated receptor gamma coactivator 1-alpha (PGC-1α) expression (160), and PGC1-α has been suggested to mediate myonuclear accretion in response to endurance exercise, which may provide transcriptional output to support mitochondrial biogenesis as part of endurance exercise-induced muscle adaptations (161). In fact, other polyphenols such as quercetin may modulate satellite cell responses through cited mechanisms, namely stimulation of SIRT1 (162) and Akt signaling pathways (163), as shown in *in vitro* experiments (164). It is interesting to note that caloric restriction is well-demonstrated to induce SIRT1 activation, which may explain the paradoxical “anabolic” effects of long-term caloric restriction when combined with exercise. Particularly, a recent comprehensive report observed that caloric restriction rejuvenates load-induced myofiber growth in a rat model of heart disease, which was caused by an increase in myonuclear accretion after overload (165).

**Figure 4:**
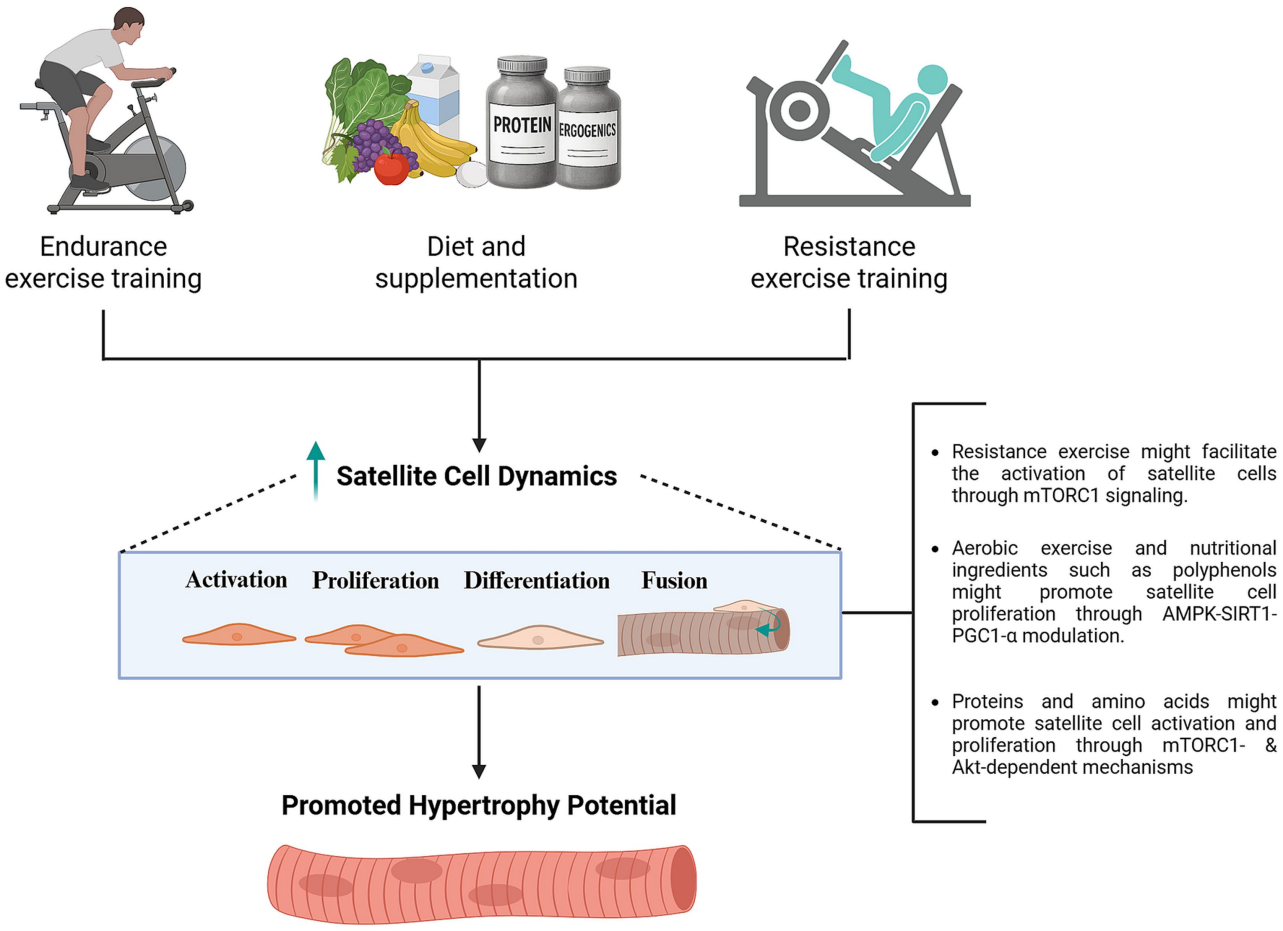
Potential effects of nutrition in combination with exercise in promoting satellite cell dynamics to support sustained hypertrophy. While mechanisms involved remain speculative, proteins and amino acids might promote satellite cell activation and proliferation through IGF-1-Akt-mTORC1 signaling mechanisms (169). Activation of SIRT1-PGC1-α pathways, among different mechanisms, might contribute to the potential effects of polyphenols in enhancing satellite cell proliferation (151). Further research is needed to better understand mechanisms involved in the modulation of satellite cell dynamics by different nutritional and ergogenic ingredients. Created with BioRender.com.

Last, different nutritional compounds with special importance for athletes’ health and performance, such as vitamin D may contribute to myonuclear accretion. For instance, *in vitro* experiments showed that administration of 10 nmol of the active form of vitamin D3, 1α,25(OH)_2_D_3,_ on human isolated skeletal muscle cells elevates myonuclear number while decreasing myonuclear domain, which was proposed to partially explain the beneficial effects of vitamin D supplementation on recovery from damaging exercise (166). In should also be noted that mixed evidence has been reported with combinations of several cited ingredients, with a recent trial failing to identify significant changes in mentioned precursor cell and myonuclear outcomes following supplementation with a multi-ingredient supplement (whey protein, creatine, leucine, calcium citrate, and vitamin D) throughout a 10-week resistance training protocol (167). Naturally, further research is needed to better understand the role of nutrition in satellite cell dynamics and myonuclear physiology, mechanisms involved, and its potential repercussions for skeletal muscle memory (Figure 4).

## 5 Conclusion

Whether skeletal muscle fully returns to a “true” baseline state following disuse or detraining remains uncertain. However, accumulating evidence supports the existence of a long-lasting “muscle memory” effect, whereby previously hypertrophied muscles exhibit an enhanced capacity for regrowth upon retraining. These effects may be driven by different mechanisms being myonuclear permanence and epigenetic modulation the most well-supported to date. Specifically, the potential permanence or long-term retention of myonuclei during moderate disuse might support transcriptional output to facilitate muscle adaptations during retraining. This has practical implications for athletes undergoing extended periods of inactivity such as recovery from injury, as it may accelerate the restoration of muscle mass and function. However, evidence suggests that aged individuals might have compromised capability to recruit satellite cells in response to exercise and, potentially, impaired retention of myonuclear content during disuse. Consistent exercise practice coupled with nutritional strategies leveraged by athletes has been suggested to facilitate satellite cell responses and myonuclei fusion in these individuals. Translating these approaches to older populations, especially to counteract the preferential atrophy of type II fibers, may offer a promising avenue for preserving muscle mass and regenerative capacity with aging.
